# A Novel Entropy-Based Centrality Approach for Identifying Vital Nodes in Weighted Networks

**DOI:** 10.3390/e20040261

**Published:** 2018-04-09

**Authors:** Tong Qiao, Wei Shan, Ganjun Yu, Chen Liu

**Affiliations:** 1 School of Economics and Management, Beihang University, Beijing 100191, China; 2 Key Laboratory of Complex System Analysis and Management Decision, Ministry of Education, Beijing 100191, China; 3 Business School, University of Shanghai for Science and Technology, Shanghai 200093, China

**Keywords:** complex network, vital nodes, weighted networks, centrality, entropy-based centrality

## Abstract

Measuring centrality has recently attracted increasing attention, with algorithms ranging from those that simply calculate the number of immediate neighbors and the shortest paths to those that are complicated iterative refinement processes and objective dynamical approaches. Indeed, vital nodes identification allows us to understand the roles that different nodes play in the structure of a network. However, quantifying centrality in complex networks with various topological structures is not an easy task. In this paper, we introduce a novel definition of entropy-based centrality, which can be applicable to weighted directed networks. By design, the total power of a node is divided into two parts, including its local power and its indirect power. The local power can be obtained by integrating the structural entropy, which reveals the communication activity and popularity of each node, and the interaction frequency entropy, which indicates its accessibility. In addition, the process of influence propagation can be captured by the two-hop subnetworks, resulting in the indirect power. In order to evaluate the performance of the entropy-based centrality, we use four weighted real-world networks with various instance sizes, degree distributions, and densities. Correspondingly, these networks are adolescent health, Bible, United States (US) airports, and Hep-th, respectively. Extensive analytical results demonstrate that the entropy-based centrality outperforms degree centrality, betweenness centrality, closeness centrality, and the Eigenvector centrality.

## 1. Introduction

The extensive applications of centrality in complex networks bring considerable value in a large number of scenarios, such as identifying the most influential spreaders in online communities [1], carrying out online precision marketing by identifying the productive and influential bloggers [2], supervising exceptional events [3], detecting the influential criminals [4], predicting essential proteins [5,6,7,8,9,10], quantifying the academic influence of scientists on the basis of co-authorship networks constructed by their publications and citations [11,12,13], discovering financial risks (for example the DebtRank algorithm first proposed by Battiston et al. [14] and then developed by Tabak et al. [15]), forecasting career movements that include promotion and resignation by analyzing the characteristics of the employees’ networks [16,17,18], and improving the robustness of power grids in order to prevent catastrophic outages [19,20,21,22]. 

In the literature, the power of an actor in a given network is mostly influenced and mirrored by the topological structure of the network [23,24,25,26]. As a result, the vast majority of widely applied centrality methods exclusively consider the topographic properties of a given network. In other words, the concept of structural centralities is designed with the purpose of detecting structural information and characterizing its influence. As noted by Lü [27], we categorize structural centralities into path-based and neighborhood-based centralities and then describe the state of art.

From the perspective of influence propagation, two significant features that most vital nodes share are propagating speed and propagating range, which should be affected by traffic flows to a great extent. Base on this idea, several classical approaches have been proposed, including betweenness centrality [23], Katz centrality [28], closeness centrality [29,30], and eccentricity [31]. Both eccentricity and closeness centrality count geodesic routes based on the idea that the efficiency of information dissemination can be maximized along the shortest paths. Yet, the value of eccentricity solely relies on the maximum length of the shortest path. By comparison, the closeness centrality takes all the shortest distances into consideration, and the value of closeness centrality can be interpreted mathematically as the inverse of the mean length of information dissemination. The betweenness centrality is a strong indicator that reflects the controllability of traffic flow. That is, most vital nodes often act like bridges that connect various communities. Generally speaking, the concept of closeness centrality indicates accessibility, and the concept of betweenness centrality shows controllability. Katz centrality [28] takes all paths into consideration and assigns less weights on the longer paths. Besides the path-based centralities mentioned above, the information centrality index [32] assumes that the loss of information in the process of propagation depends on the length of the path. Therefore, this approach calculates the quantity of information contained in all potential traffic flows.

The extensive studies of the neighbor nodes lay the foundation for neighborhood-based centralities. The LocalRank algorithm proposed by Chen et al. [33] not only utilized the information contained in the immediate neighbors of a given node but took into account the fourth-order neighbors. Gao et al. [34] extended the algorithm to weighted networks. However, it is clear that LocalRank and its revised version fail to capture the process of influence propagation. As was noted by Petermann [35], the local interconnectedness inevitably affects the process of the information transmission. In light of that thought, Chen et al. [36] analyzed the role of clustering and proposed the ClusterRank algorithm by considering the effect of the clustering coefficient on the spreading speed. As a matter of fact, the nodes with high values of ClusterRank are usually the nodes that belong to distinct communities. Thus, there is acceleration of propagation once information passes through those nodes. The centralities discussed above ignore the significance of the location of a node in a given network. Kitsak et al. [37] believed that coreness could be a more effective index to distinguish the relative importance of nodes. Zeng et al. [38] and Pei et al. [39] applied the k-core algorithm to large real-world networks. However, the original k-core algorithm may result in plenty of indistinguishable nodes with same value of coreness. Moreover, the initial algorithm exclusively makes use of the residual degree. Thus, many researchers [40,41,42,43] proposed many modified k-core algorithms from different point of views. The last representative neighborhood-based structural centrality is the H-index [44], a local centrality initially employed to measure researchers’ academic influence by counting their publications [45,46]. Recently, Lü et al. [47] proved the convergence of the *H*-indices. 

Besides the structural centralities discussed above, recently entropy theories have been applied to measure the complexity and uncertainty of complex networks. In [48,49,50,51,52,53,54], the authors have demonstrated that better results of quantitative analyses of influence can be obtained by using entropy theory. In our previous work [55], the same conclusion has been drawn that defined entropy centrality has proven far superior to other widely used methods, such as degree centrality, betweenness centrality, closeness centrality, Eigenvector centrality, and PageRank. It is clear that the ideal algorithm, free of any limitations or assumptions, does not exist. The previous model was intentionally applicable to undirected, unweighted networks only and not general cases. 

In this paper, with the intention of providing a more effective and more general framework to quantify the power of each actor in a directed, weighted network, we first study the properties of directed, weighted networks. Then, by generalizing the features of two-way behaviors between two actors, we make direct use of directed networks to quantify influence. In particular, the total power of each actor in a given network can be calculated as the result of integrating the direct influence on its one-hop neighbors and the indirect influence on its two-hop neighbors. In terms of the direct influence mentioned above, we divide it into two parts measured by the structural entropy and the interaction frequency entropy, respectively. Generally, the former kind of information entropy reflects the communication activity, popularity, and strength of an actor, and the latter kind of information entropy, which deep mines the information carried by weights corresponding to the interaction frequency, mirrors the closeness among an actor and its neighbors. Moreover, we adopt the two-hop subnetworks to capture the process of influence propagation. In order to evaluate the effectiveness of the entropy centrality, we conduct experiments on four weighted networks including adolescent health, Bible, United States (US) airports and Hep-th. We also compare the performance of the entropy-based centrality with that of degree centrality, betweenness centrality, closeness centrality, and the Eigenvector centrality. Extensive experimental results prove that the proposed method has an obvious advantage in identifying influential nodes. 

## 2. Model Description

As discussed above, in our previous work [55], we proposed a centrality method, which depicts the connections among node pairs by using Shannon’s entropy and characterizes the process of influence spread by using two-hop subnetworks. However, the proposed method could be inappropriate in the case of directed and weighted networks. Thus, we modified the algorithm and extended its application scenarios. 

Now, let us consider a directed, weighted network graph G(V,E,W), where V denotes the set of vertices in a given network, E represents the set of directed edges from one node to another, and set W corresponds to the weighted values. For instance, set V of vertices corresponds to individual users in online social network, set E represents the traffic flow among users i–j, and set W of weighted values indicates the total number of any kinds of messages sent from user i–j. Examples abound in real-world networks. 

**Figure 1:**
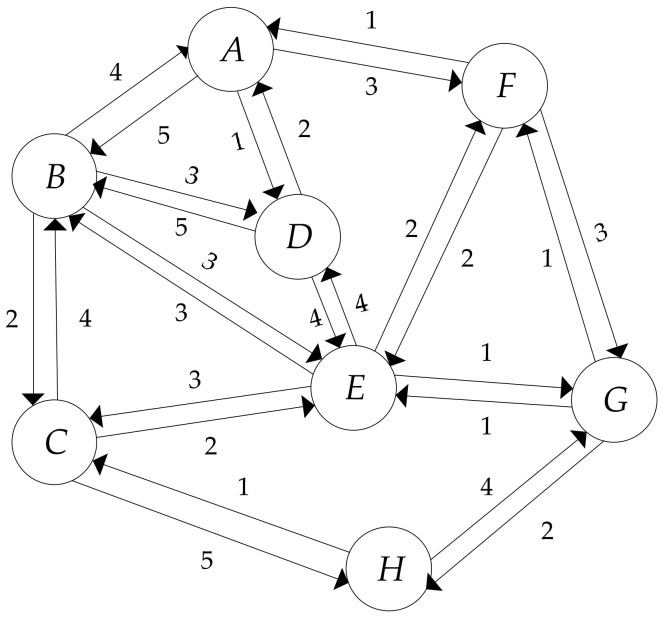
An example of a directed, weighted network.

With the purpose of describing the definition of the entropy centrality, we use a simple, directed, weighted network as an example, and the graph can be seen in Figure 1. In this simple network, each node corresponds to an airport. Each directed edge represents an airline from one airport to another. Correspondingly, the weight of each directed edge indicates the total number of flights on that connection in the given direction. The values of weights, denoted as $C_{ij}$, are listed in Table 1. 

**Figure 2:**
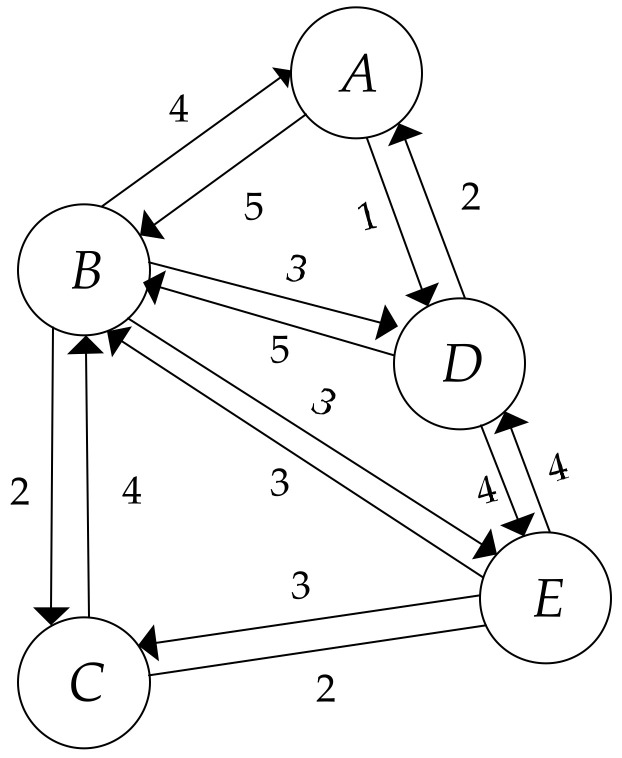
The subgraph constructed by node B and its neighbor nodes.

In this paper, we deconstruct the global power of a node into two parts, including its local power and its indirect power. The local power of a node indicates its accessibility, activity, popularity, and strength in the small world to which it belongs. Inspired by that thought, first, we deconstruct a complete network into several serval subnetworks centered on certain nodes. For example, Figure 2 shows the subgraph centered on node B, denoted as $subgraph_{B}$. 

Then, we observe that each subnetwork contains various kinds of information that may be useful in the process of quantifying the local power, such as the in-degree centrality, out-degree centrality, and weights of edges. For instance, considering a random online social network, if user i follows user j, consequently there will be a directed link from node i to j. Thus, the out-degree centrality of a node reflects its social activity or its authority, and the in-degree centrality of a node interprets its social popularity. So, it seems more suitable to combine multiple information contained in different centralities. In light of that idea, we would like to introduce the following definition of the subgraph degree centrality.

Given a directed weighted graph G(V,E,W), for a vertex $v_{i}\in V$, the i-centered subgraph represented by $G_{i}$ can be built by node i and its neighbors. Accordingly, the subgraph degree centrality of node i and its neighbor j, denoted as $SDC_{i}$, equals the summation of its in-degree centrality and its out-degree centrality, namely

(1) $$SDC_{i}=DC_{i}^{in}+DC_{i}^{out}$$

where $DC_{i}^{in}$ represents the in-degree centrality of node i in the given subgraph (i.e., the number of nodes having a directed link pointing to node i in the given subgraph) and $DC_{i}^{out}$ indicates the out-degree centrality of node i in the given subgraph (i.e., the number of directed edges from node i to other nodes in the given subgraph). Let us take Figure 2 as an example. In this particular subgraph, we count both in-degree and out-degree for each node on the basis of Equation (1). The results can be seen in Table 2.

Notice that Figure 2 illustrates two-way behaviors between node pairs. Clearly, it is a special case. Then, one may ask what the value of subgraph degree centrality will be if only one-way connection is showed (for example, in the citation network, a node may only have the in-degree centrality or the out-degree centrality). In that situation, the subgraph degree centrality is identical to the in-degree centrality or the out-degree centrality. As for the undirected, weighted networks, the subgraph degree centrality of a specific node should be the number of nodes connecting to the given node in the given subgraph. Notably, Equation (1) is also suitable for other types of networks, such as online social networks, email communication networks, collaboration networks, internet networks, etc.

In order to quantify the local power of a given node, we combine the advantages of both topological structure and information entropy. We believe that more precise influence ranking results will be obtained when information is properly utilized. Therefore, we propose a novel definition of entropy centrality, which takes both structural entropy and frequency entropy into consideration. The structural entropy, which takes advantage of topographic properties of the subgraph, evaluates the activity, popularity, and strength of a given node in specific subnetwork. The frequency entropy, which makes use of information contained in the weights of directed links, depicts the accessibility of a given node. 

Based on the concept of subgraph degree centrality, the structural information entropy $I_{i}^{s}$ for node *i* in subgraph $G_{i}$ is defined as follows:

(2) $$I_{i}^{s}=-\overset{M+1}{\underset{i=1}{\sum }}\frac{SDC_{i}}{\sum_{i}^{M+1}SDC_{i}}log\frac{SDC_{i}}{\sum_{i}^{M+1}SDC_{i}}$$

where M denotes the number of neighbors of node i in subgraph $G_{i}$. 

Generally, the weight of directed links acts as an effective indicator that reflects the interaction frequency. We believe that close relationships between node pairs is mainly maintained by their frequent interactions. Motivated by that, the definition of the interaction frequency entropy of node i, denoted as $I_{i}^{f}$, in subgraph $G_{i}$ is stated as follows:

(3) $$I_{i}^{f}=-\overset{M}{\underset{j=1}{\sum }}\frac{C_{ij}}{\sum_{k=1}^{M}C_{ik}}log\frac{C_{ij}}{\sum_{k=1}^{M}C_{ik}}$$

where M indicates the total number of node i’s neighbors and $C_{ij}$ denotes the weight of a directed edge in the given direction. Notably, $C_{ij}$ in the Equation (3) should be replaced by the weights of the undirected edges, denoted as $W_{ij},$ for the undirected weighted networks. As explained above, the interaction frequency entropy of a given node indicates its accessibility to some extent. 

In line with Equations (2) and (3), the local influence of node i on its one-hop neighbors, denoted as $LI_{i},$ equals the summation of the structural information entropy, denoted as $I_{i}^{s},$ and the interaction frequency entropy, denoted as $I_{i}^{f},$ multiplied by two coefficients respectively, namely

(4) $$LI_{i}=\omega_{1}I_{i}^{s}+\omega_{2}I_{i}^{f}$$

where $\omega_{1}$ and $\omega_{2}$ represent the weight coefficients, respectively, and $\omega_{1}+\omega_{2}=1$.

In our previous work [55], we were enlightened by the inspiring work done by Christakis [56,57] and other researchers [58,59,60,61] who found that meaningful influence can no longer be detectable beyond the boundary of three or four degrees. Consequently, we choose two-hop subnetworks to capture how influence propagates through the whole network. The key assumption of the two-hop influence propagating model is that we might not influence nor be influenced by people at three degrees and beyond. Consistent with analytical results by the empirical study of both artificial datasets and real-world datasets, the superiority of the previous method has been proven in comparison with other widely used approaches, such as degree centrality, betweenness centrality, closeness centrality, the Eigenvector centrality, and PageRank. In addition, the two-hop subnetwork used to measure the indirect power of a given node is concise. Accordingly, in this paper we adopt the same model to describe the process of influence propagation.

**Figure 3:**
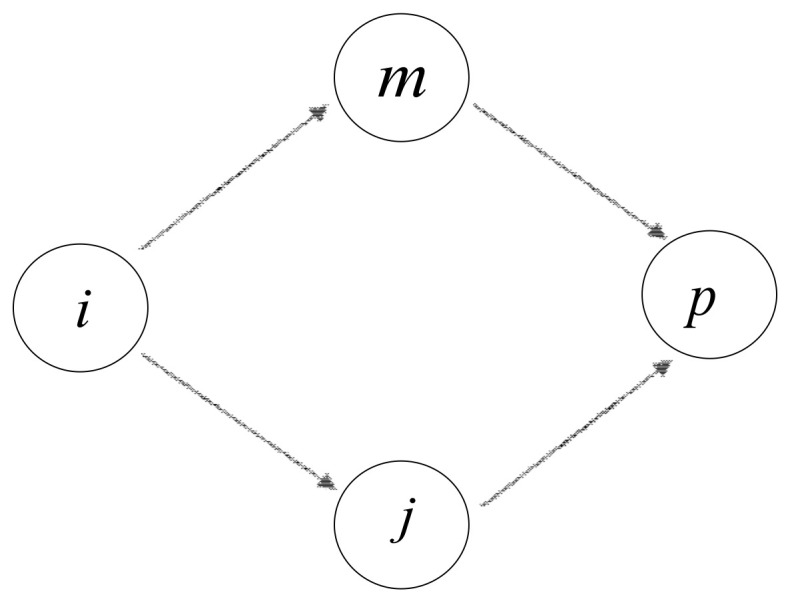
Double path.

Suppose that node p is one of two-hop neighbors of node i, and node j is one of the common neighbors between node i and p. Let $N_{ip}$ represent the number of common one-hop neighbor nodes between i and p. Let us take $N_{ip}=2$ for example, which can be seen in Figure 3. Notice that two traffic flows exist from node i–p. As discussed above, we have already calculated the local power of each individual node. Consequently, the indirect influence of node i on its two-hop neighbor node p, denoted as $II_{ip}$, is stated as follows

(5) $$II_{ip}=\frac{LI_{i}\times LI_{j}+LI_{i}\times LI_{m}}{2}$$

where $LI_{i}$, $LI_{j},$ and $LI_{m}$ denote the local power of node i, j, and m, respectively. 

Consistent with the above analysis, the indirect influence of node i on its two-hop neighbor p, denoted as $II_{ip}$, is stated as follows:

(6) $$II_{ip}=\overset{N_{ip}}{\underset{p=1}{\sum }}\frac{LI_{i}\times LI_{j}}{N_{ip}}$$

where $N_{ip}$ represents the total number of the common one-hop neighbors between node i and p.

Evidently, the indirect influence on its two-hop neighbors, denoted as $II_{i},$ is stated as follows:

(7) $$II_{i}=\frac{\sum_{p=1}^{M_{i}}II_{ip}}{M_{i}}$$

where $M_{i}$ corresponds to the number of two-hop neighbors of node i.

Eventually, the total power of node i in the given network G(V,E,W) represented by $I_{i}$ equals the summation of the direct influence, denoted as $LI_{i},$ and the indirect influence, denoted as $II_{i},$ multiplied by two coefficients, respectively, namely

(8) $$I_{i}=\theta_{1}LI_{i}+\theta_{2}II_{i}$$

where $\theta_{1}$ and $\theta_{2}$ stands for the weight of local influence $LI_{i}$ and indirect influence $II_{i}$, and correspondingly, $\theta_{1}+\theta_{2}$ = 1.

We use the same network shown in Figure 1 as an example to describe the calculating process of the proposed algorithm. On the basis of Equation (1), the subgraph degree centrality, denoted as SDC, in $G_{B}$ is listed in Table 2. In light of the values of SDC shown in Table 2, if 10 is the base of the logarithmic function, then the structural entropy of node B is computed as follows:

(9) $$I_{B}^{s}=-\overset{5}{\underset{i=1}{\sum }}\frac{SDC_{B}}{\sum_{i}^{4+1}SDC_{i}}log\frac{SDC_{B}}{\sum_{i}^{5}SDC_{i}}=0.6836$$

Furthermore, following Equation (3), the interaction frequency entropy is expressed as follows:

(10) $$I_{B}^{f}=-\overset{4}{\underset{j=1}{\sum }}\frac{C_{ij}}{\sum_{k=1}^{4}C_{ik}}log\frac{C_{ij}}{\sum_{k=1}^{4}C_{ik}}=0.5898$$

We purposely applied the same sets of coefficients that were used in [54,55]. The authors have demonstrated that the entropy-based centrality outperforms the classic degree-based centralities and path-based centralities under the conditions of this particular set of parameters. By introducing this particular set of coefficients, the resulting value of influence will always be between zero and one. 

Particularly, we set $\omega_{1}=0.4$ and $\omega_{2}=0.6$ and consequently redefined the local influence of node B, represented by $LI_{B}$, which is stated as follows:

(11) $$LI_{B}=0.4I_{B}^{s}+0.6I_{B}^{f}=0.6273$$

In accordance with Equations (6) and (7), the indirect influence of node B on its two-hop neighbors, denoted as $II_{B},$ is given as follows:

(12) $$II_{B}=\left(LI_{B}\times LI_{E}+\left(LI_{B}\times LI_{A}+LI_{B}\times LI_{E}\right)/2+LI_{B}\times LI_{C}\right)/3=0.3713$$

In particular, we set $\theta_{1}=0.6$ and $\theta_{2}=0.4$, and as a result, $I_{B}$ corresponding to the total influence of node B in the given network is described as follows:

(13) $$I_{B}=0.6LI_{B}+0.4II_{B}=0.5249$$

As discussed above, based on entropy centrality, the power of each node and the corresponding ranking results are recorded in Table 3 and Table 4, respectively.

## 3. Performance Evaluation

In order to evaluate the performance of our proposed ranking algorithm, we use four real-world networks, which consist of two directed weighted networks and two undirected weighted networks. The four real-world networks are: (i) Adolescent health [62], a directed network with positively weighted edges. The data was generated from a survey conducted in 1994/1995. In that network, each node corresponds to a student who was asked to list his/her five best male friends and five best female friends. The directed edge between student i and j, denoted as $e_{ij},$ represents that student i chooses student j as his/her friend. Furthermore, higher weighted values indicate more interactions; (ii) (US) airports [63], a directed network of US infrastructure in 2010. Each node corresponds to an airport and the directed edge represents the airline connection from one airport to another. The weighted value shows the total number of flights on that connection in the given direction; (iii) Bible [63], an undirected network containing nouns (places and names) in the King James version of the Bible and information about their co-occurrences. A node represents one of the above noun types, and an edge indicates that two nouns appeared together in the same Bible verse. The weight of edge denotes how often two nouns occurred together; (iv) Hep-th [64], an undirected, weighted collaboration network with nodes corresponding to scientists posting preprints on the Hep-th and edges indicating collaborations. The assigned weights, which reflect the strength of collaborative ties can be obtained based on the number of preprints that each pair of researchers has done together and the number of other coauthors they worked with on each of those preprints. Table 5 shows the statistics of the four weighted networks mentioned above. 

In this paper, we apply the susceptible-infectious model to characterize the dynamic process of influence propagation. In the SI model, all nodes apart from the infected nodes are susceptible initially. A susceptible node will be infected by its infected neighbor nodes with probability β. However, in terms of the weighted networks, the infection probability is not a constant. The information contained in the directed edges should also be considered. In the literature, Yan et al. [65] defined the infection probability, denoted as $\lambda_{ij}$, as $\lambda_{ij}={\left(\frac{w_{ij}}{w_{max}}\right)}^{\alpha },$ in which susceptible node i comes in contact with its infected neighbor node j and gets infected. Where α corresponds to a constant with a positive value, $w_{ij}$ is the weighted value of the directed edge $e_{ij}$, and $w_{max}$ denotes the largest value of $w_{ij}$. In addition, Wang et al. [66] introduced another kind of infection transmission. The probability that susceptible node i is infected by its neighbor i is stated as $1-{\left(1-\beta \right)}^{w_{ij}}$, where β is a positive constant and $w_{ij}$ denotes the weighted value on the edge in the given direction. In this paper, we adopt the latter form of infection transmission proposed by Wang et al. [66]. 

**Figure 4:**
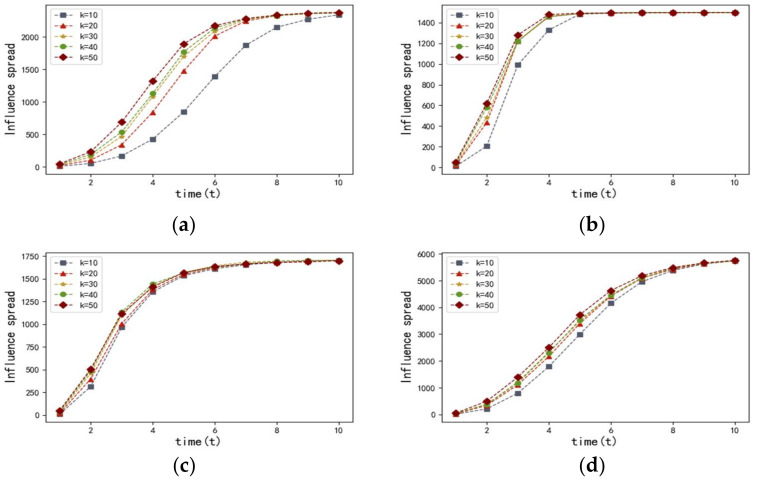
The influence spread with different k at time t. The results are obtained by using the entropy-based centrality in the four weighted networks including: (**a**) adolescent health; (**b**) US airports; (**c**) Bible, and (**d**) Hep-th, respectively.

**Figure 5:**
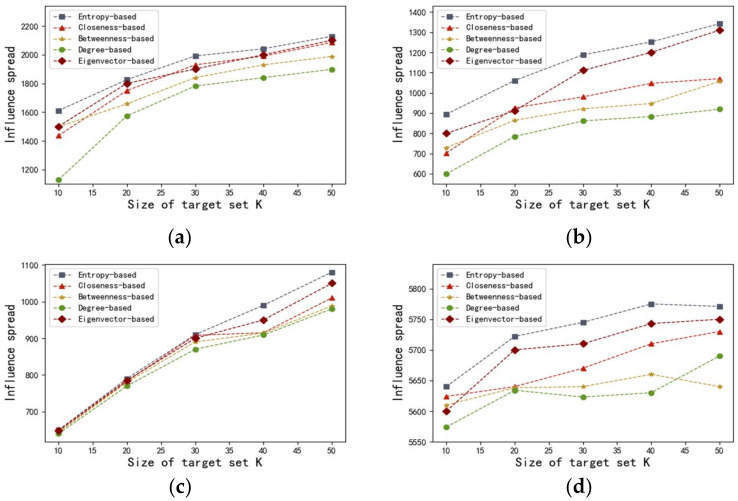
The influence spread with different k in the four weighted networks including: (**a**) adolescent health, (**b**) US airports, (**c**) Bible, and (**d**) Hep-th, respectively. The results are obtained by using the entropy-based centrality, degree centrality, betweenness centrality, and closeness centrality, respectively.

In order to evaluate the efficiency of proposed method, we use the entropy-based centrality to select k nodes with most influential as seed nodes. In comparison, we also test the performances of degree centrality, betweenness centrality, closeness centrality, and the Eigenvector centrality. Then, the influence spread can be seen as an indicator of the algorithm’s effectiveness. In order to obtain that index, we run the SI simulation on four networks 1000 times and select the mean value of the influence spread. Initially, we set the value of k as (10,20,30,40,50). Correspondingly, the results are illustrated in Figure 4 and Figure 5. 

Figure 4 describes the influence spread of the proposed entropy-based centrality model with different k at time t in the four networks discussed above. The results of the four networks, which are shown in Figure 4, have proven that there is a positive correlation between the influence spread and the value of set k. We observed that the more most influential nodes there are, the more nodes can be influenced. It is also worth noting that the speed of infection transmission accelerates as the value of set k increases.

Figure 5 illustrates the influence spread of various centralities with different k sets of influential seeds for the four networks. As shown in Figure 5, in terms of all the networks, degree centrality, which belongs to the neighborhood-based centralities, preforms badly. One possible reason is that degree centrality, which exclusively takes immediate neighbors’ information into consideration, is no longer applicable to capture the process of influence propagation. Even though betweenness centrality performs better than degree centrality, the effectiveness of betweenness centrality is significantly inferior to that of closeness centrality and the Eigenvector centrality, as well as that of the entropy-based method we proposed. One plausible explanation is that supposing a large number of nodes are not contained in other node pairs’ shortest paths, the values of betweenness should be zero, leading to many indistinguishable nodes with the same betweenness. Compared with closeness centrality, our entropy-based approach can obtain better results in the networks of adolescent health, US airports, and Hep-th, because the same k quantity of initial seeds sorted by entropy centrality eventually infected more nodes. Notice that the curves generated from closeness centrality and the entropy-based centrality are almost overlapping in the Bible network, which indicates that these two centralities show similar efficiency. As the value of k increases from 40 to 50, many more nodes have been infected by the initially infected nodes obtained by applying entropy centrality. Also, it is obvious that our proposed entropy centrality performs better in the networks of adolescent health, US airports, and Hep-th, because the nodes with higher entropy-based centrality, if infected, more quickly infect many more nodes in the networks in comparison with the Eigenvector centrality. In the Bible network, our proposed algorithm achieves better results than the Eigenvector centrality as the value of k increases from 30 to 50. Based on the results illustrated in Figure 5, it can be concluded that the Eigenvector centrality is more effective in identifying vital nodes in comparison with closeness centrality, at least in these four cases.

Figure 5 shows that the influence spread respectively corresponding to the entropy-based centrality and the Eigenvector centrality significantly increases as the value of k varies from 10 to 50. Finally, these two curves come closer and closer. In addition, identical sets of influential nodes (infected seeds) sorted by the entropy-based centrality infect many more susceptible nodes compared with those sorted by the other four centralities. In addition, the influence spread of the proposed model rises more significantly and quickly in comparison with that of other four centralities. In short, it can be concluded that the entropy-based centrality has an advantage in detecting influential spreaders and performs best in all four weighted real-world networks, namely adolescent health, US airports, Bible, and Hep-th. In fact, the performance of the Eigenvector centrality is the second best in the four weighted networks.

## 4. Conclusions and Discussion

In this paper, with the purpose of identifying the most influential spreaders, we propose a novel definition of entropy-based centrality. By studying the topological properties of the complex network, we introduce structural entropy and interaction frequency entropy. In general, by combining the advantages of the topological structure and information entropy, this entropy-based centrality can not only make full use of the information contained in neighbor nodes but also quantify influence from the perspective of information spreading. In order to verify the efficiency of the entropy-based centrality, we used four weighted real-world networks with varied instance sizes, degree distributions, and densities, including two directed networks and two undirected networks. When using the SI model, the entropy-based centrality performed best when compared with degree centrality, betweenness centrality, closeness centrality, and the Eigenvector centrality on the basis of extensive analytical results.

It is also noteworthy that there are similarities and differences between the proposed method and other structural centralities. For example, both the proposed approach and degree centrality are based on the idea that the power of a given node can be reflected by its capacity to influence the behaviors of its surrounding neighbors. However, degree centrality fails to capture the process of influence propagation compared with the proposed method. Both ClusterRank [36] and the proposed method take the number of immediate neighbors into consideration. However, the ClusterRank algorithm [36] uses clustering coefficients to describe the information spreading process. Both the proposed method and the information index [32] are built on the assumption that information will be lost during every hop in the network, and therefore, the longer the path, the greater the loss. However, the information index considers information contained in all possible paths from a given node to others. Unlike the proposed method, closeness centrality [29,30], betweenness centrality [23], and eccentricity [31] compute the influence of a given node by measuring the shortest paths. In the literature, the mapping entropy [52], which is fully based on the local information contained in a given node and its immediate neighbors, fails to capture the propagating process. In comparison with the proposed method, which is established on the basis of information entropy, another kind of entropy-based centrality proposed by Fei and Deng [53] takes advantage of relative entropy and the TOPSIS method. They also treat centralities from various measures as multiple attributes in the process of quantifying influence. 

However, it is clear that perfect algorithms, free of any limitations or assumptions, do not exist. There is still space to improve our entropy-based centrality. Firstly, in the calculation process, we find that the entropy-based centrality values of some nodes are extremely small and consequently indistinguishable. Secondly, the model of determining direct influence is highly neighborhood-based, resulting in a few indistinguishable nodes with the same entropy-based centrality. Thirdly, the entropy-based centrality is no longer applicable to the networks with negatively weighted edges because the base of the logarithm function, which is the foundation of the information entropy, must be positive. However, there are still plenty of bipartite networks with both positive and negative edges, such as Epinions trust, Wikipedia conflict, Chess, Wikipedia elections, and so on. Accordingly, researchers suggest plenty of algorithms, for instance, the group-based ranking approach proposed by Gao et al. [67] to measure a user’s reputation, the correlation-based iterative method [68], the iterative algorithm with reputation redistribution [69], and the HITs method for bipartite networks [70].

As for future work, we expect to further improve the entropy-based centrality. We are also looking forward to applying the entropy-based centrality in the real world, such as in delivering advertisements for companies, predicting career movements, constructing the recommender systems, studying the interdisciplinary knowledge network of China [71] and evaluating airports in China. 

## Figures and Tables

**Table 1:** The number of airline connections from one airport to another.

Between Two Airports	The Number of Airlines	Between two Airports	The Number of Airlines
A→B	5	E→B	3
A→D	1	E→C	3
A→F	3	E→D	4
B→A	4	E→F	2
B→C	2	E→G	1
B→D	3	F→A	1
B→E	3	F→G	3
C→B	4	F→E	2
C→E	2	G→E	1
C→H	5	G→F	1
D→A	2	G→H	2
D→B	5	H→C	1
D→E	4	H→G	4

**Table 2:** The results of $SDC_{i}$ of nodes in subgraph $G_{B}$.

Node	$DC_{i}^{in}$	$DC_{i}^{out}$	$SDC_{i}$
*B*	4	4	8
*A*	2	2	4
*C*	2	2	4
*D*	3	3	6
*E*	3	3	6

**Table 3:** The results of the redefined entropy centrality of each node.

Node	Local Influence	Indirect Influence	Total Influence
*A*	0.4736	0.2625	0.3892
*B*	0.6273	0.3713	0.5249
*C*	0.4955	0.3080	0.4205
*D*	0.5073	0.3283	0.4357
*E*	0.6956	0.3619	0.5521
*F*	0.4930	0.2915	0.4124
*G*	0.5004	0.2987	0.4197
*H*	0.3110	0.2323	0.2795

**Table 4:** The ranking results.

Node	No.
*E*	1
*B*	2
*D*	3
*C*	4
*G*	5
*F*	6
*A*	7
*H*	8

**Table 5:** The basic statistics of the four weighted networks.

Networks	n	m	c ^1^	AD ^2^
Adolescent health	2539	12,969	14.2%	10.216 (overall)
US airports	1574	28,236	38.4%	35.878 (overall)
Bible	1773	9131	16.3%	18.501
Hep-th	8361	15,757	32.7%	3.768

^1^ c denotes the clustering coefficient. ^2^ AD represents average degree.
